# A Fast Response−Recovery 3D Graphene Foam Humidity Sensor for User Interaction

**DOI:** 10.3390/s18124337

**Published:** 2018-12-08

**Authors:** Yu Yu, Yating Zhang, Lufan Jin, Zhiliang Chen, Yifan Li, Qingyan Li, Mingxuan Cao, Yongli Che, Junbo Yang, Jianquan Yao

**Affiliations:** 1 Department of Electrical and Electronic Engineering, South University of Science and Technology of China, Shenzhen 518055, China; yuyu1990@tju.edu.cn (Y.Y.); jlfking@tju.edu.cn (L.J.); chenzl@tju.edu.cn (Z.C.); yifanli@tju.edu.cn (Y.L.); liqingyan216@163.com (Q.L.); mingxuancao@tju.edu.cn (M.C.); cheyongli@tju.edu.cn (Y.C.); jqyao@tju.edu.cn (J.Y.); 2 Key Laboratory of Opto-Electronics Information Technology, Ministry of Education, School of Precision Instruments and Opto-Electronics Engineering, Tianjin University, Tianjin 300072, China; 3 Center of Material Science, National University of Defense Technology, Changsha 410073, China; yangjunbo008@sohu.com

**Keywords:** three-dimensional graphene foams, humidity sensor, fast response, user interaction

## Abstract

Humidity sensors allow electronic devices to convert the water content in the environment into electronical signals by utilizing material properties and transduction techniques. Three-dimensional graphene foam (3DGF) can be exploited in humidity sensors due to its convenient features including low-mass density, large specific surface area, and excellent electrical. In this paper, 3DGF with super permeability to water enables humidity sensors to exhibit a broad relative humidities (RH) range, from 0% to 85.9%, with a fast response speed (response time: ~89 ms, recovery time: ~189 ms). To interpret the physical mechanism behind this, we constructed a 3DGF model decorated with water to calculate the energy structure and we carried out the CASTEP as implemented in Materials Studio 8.0. This can be ascribed to the donor effect, namely, the electronic donation of chemically adsorbed water molecules to the 3DGF surface. Furthermore, this device can be used for user interaction (UI) with unprecedented performance. These high performances support 3DGF as a promising material for humidity sensitive material.

## 1. Introduction

Humidity sensors have aroused attention in many fields such as industry, agriculture, and environment [1,2], and medical devices [3]. Generally, they measure humidity through a variety of transduction techniques, including the use of resistive [4,5], capacitive [6], optical fiber [7], and field effect transistors [8,9]. There are also some high precision impedance-frequency transducers using quartz crystals which compensate temperature drift, and have fast response, as investigated by in Matko et al. [10]. In high air humidity measurement there is a problem with response time of the sensors in conventional methods. A solution for this problem is sensors for high air humidity measurement which use open capacitors with very low response time such as is described by Vojko et al. [11].

As an active material for absorbing water molecules, a series of sensing materials including polymers [12], metal oxides [8], carbon nanotubes [13,14], graphene dioxide [15,16], and composites [5,17] have been exploited in humidity sensors. For instance, Zhang et al. [12] described humidity sensors utilizing poly(*N*-vinyl-2-pyrrolidone) (PVP), poly(vinyl alcohol) (PVA), and hydroxyethyl cellulose (HEC). In particular, after using PVP, the humidity sensors exhibited response and recovery times between 11% and 95% relative humidity (RH) were about 37 s and 10 s, respectively. Wang et al. [8] applied a single SnO_2_ nanowire (NW) to fabricate a humidity sensor, which exhibited a wide sensor RH range (5~85%), and the response and recovery times were 120~170 s and 20~60 s, respectively. Zhao et al. [15] investigated a humidity sensor based on multi-wall carbon nanotubes where the sensor testing range was about 11% to 97% RH, the response time was 45 s, and the recovery time was 15 s. Borini et al. [15] exploited graphene oxide in a humidity sensor and obtained an unprecedented response speed (~30 ms response and recovery times) in a range of 30% to 70% RH. Zhang et al. [5] utilized a graphene oxide (GO)/poly(diallyldimethylammonium chloride) (PDDA) nanocomposite film to fabricate a humidity sensor. The humidity sensor exhibited ultrahigh performance over a wide range of 11~97% RH, and the recovery time is 125 s at 11% RH. Thus, each sensing material has its own advantages and specific conditions of application. In addition, with large surface area to volume ratio, nanomaterials are attractive to fabricate humidity sensors with ultrahigh performance features including high sensitivity and fast response times.

Recently, graphene with three dimensional (3D) architectures, including foams, networks, and gels have been investigated [18,19,20,21]. These 3D graphene-based materials not only have the characteristics of graphene, but also have high specific surface area, low density, good mechanical strength and good conductivity [22]. Because of its wide accessibility, easy synthesis and solution processability, high chemical stability and strong adaptability [23,24], 3D graphene foam (3DGF) has attracted great interest in various sensing applications. Meanwhile, 3DGFs are efficient materials for biosensors and gas-sensing devices given their low-mass density, large surface area, good mechanical stability, and high electrical conductivity. Huang and coworkers [25] synthesised 3DGF/CuO nanoflower composites as single-chip independent 3D biosensors for the electrochemical detection of ascorbic acid with outstanding biosensing properties, such as an ultrahigh sensitivity of 2.06 mA mM^−1^ cm^−2^ to ascorbic acid at a 3 s response time. Besides that, Yavari et al. [24] used macroscopic 3DGF to fabricate gas detectors with high sensitivity. Generally, these electrical-type 3DGF sensors exhibit high sensitivity due to these properties including an ultrahigh surface area, and its electronic properties. It shows a strong dependence on surface absorbents (including gas molecules), which can change the carrier density of graphene [24]. Therefore, it is necessary to develop a new type of humidity sensor based on 3DGF by utilizing the unique structure and chemical characteristics and avoiding its shortcomings.

In this paper, we fabricate a humidity field effect transistor based on 3DGF and develop test equipment to measure the properties of the device. It exhibits a high performance over a broad RH range from 0% to 85.9%, with fast response and recovery times. To interpret the physical mechanism, we construct the 3DGF model decorated with water and apply CASTEP in the Materials Studio software to calculate the energy structure. Herrin, we explore the potential of 3D GF for portable, reliable and low cost humidity sensing applications in the future.

## 2. Materials and Methods

**Figure 1:**
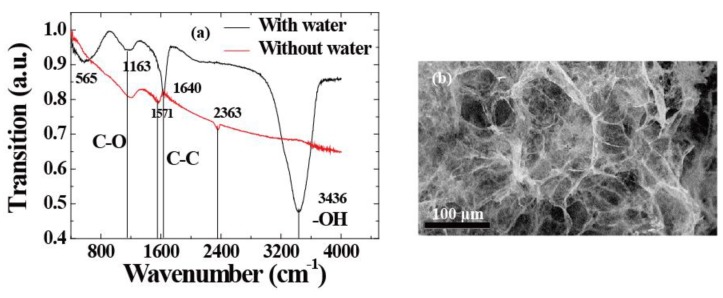
(**a**) FTIR spectra of 3D graphene with or without water molecule. (**b**) Field emission scanning electron microscopy (SEM) images of 3DGF.

Utilizing a modified Hummers’ method, [19,20,21] graphene oxide, denoted as GO, was synthesized from natural graphite powder by an oxidation reaction. GO ethanol solution (50 mL) with the concentration of 1 mg mL^−1^ was sealed in a 100 mL Teflon-lined autoclave which was then heated up to 180 °C and held for 12 h. Then the autoclave was cooled naturally to room temperature. The prepared ethanol intermediates were carefully removed from the autoclave by a slow and gradual solvent exchange with water. After the solvent exchange process was completed, the product filled with water was freeze-dried and then dried at 120 °C for 2 h in a vacuum oven. Finally, the sample was annealed at 450 °C in H_2_/Ar (5/95, *v*/*v*) for 6 h. Finally, the sample was treated in a UV ozone system for 15 min to obtain the final 3DGF. The infrared spectrum of 3DGF was recorded on a Fourier transform infrared (FTIR) spectrophotometer using potassium bromide (KBr) pellets. Figure 1a shows the FTIR spectra of three-dimensional graphene foam (3DGF)) with water molecules (black line) and dry (red line) conditions. It can be seen that a broad peak at 3436 cm^−1^ corresponds to the vibration due to the stretching and bending of OH groups present in the water molecules adsorbed by 3DGF. Thus, it was concluded that 3DGF exhibits strong hydrophilicity. Meanwhile, the absorption peaks at 565, 1163, and 1640 cm^−1^ correspond to the symmetric and antisymmetric stretching vibrations of C=O, C–O, and C–C groups for 3DGF, respectively. Figure 1b shows the surface morphologies of the 3DGF. Field emission scanning electron microscopy (SEM) images show clear, layered and interconnected three-dimensional uniform graphene sheets. It can be concluded that it forms a spongy porous network structure. [20]. The samples are cut into rectangular slabs (14 mm × 2 mm), and both sides are pasted by copper conductive adhesives on silicon substrates with a size of 14 mm × 14 mm for electrical contact.

**Figure 2:**
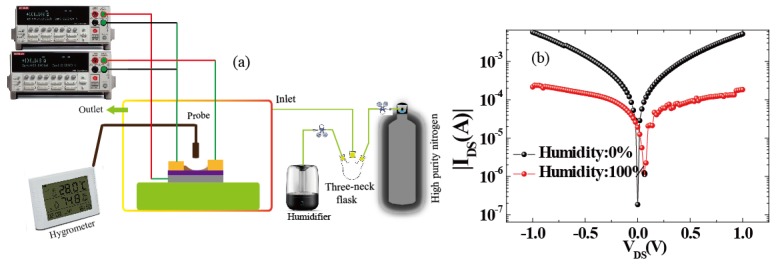
(**a**) Testing equipment used for the electrical characterization of 3DGF humidity sensors. (**b**) Output characteristic of the device decorated with or without water molecules.

For humidity sensors, chemical or physical reactions between water molecules and materials induce changes in channel current. External factors including the water concentration, temperature, and operating conditions will impact the performance of the device. For accurate measurements, as shown in Figure 2a, we used a closed box as an experimental chamber to control the humidity. In detail, the water concentrations were controlled by the ratio of saturated water vapor generated by a humidifier to high-purity nitrogen. We assure high quality humidity measurement in different ambient temperature operating conditions in climate chamber as shown in [26]. In order to measure the channel current flowing into the drain electrode (*I_DS_*) [27,28,29], the source (with ground connection) and drain electrodes were connected with a Keithley 2400 apparatus (Tektronix China Ltd, Shanghai, China). The electrical measurements were also performed with this system, and the RH of the environment was measured by a commercial humidometer. Therefore, as described by Figure 2b, the output characteristics of the device were measured under dry and humid conditions. It shows that when the RH level was fixed to 100%, the channel current (*I_DS_*) became lower than the conditions under drying. Meanwhile, the Dirac point shifted towards the positive direction. This donor effect [1] has been ascribed to the donation of electrons from the chemically adsorbed water molecules to the 3DGF. It can be concluded that the water molecules decorated in 3DGF will attract electrons and remain as holes, leading to p-type doping. Furthermore, water molecules decrease the charge mobility of 3D graphene, leading to lower currents. Through swelling or the 2D capillary effect [7,15,24], the dielectric constant will increase and the resistance decrease after adsorbing water molecules (confirmed using FTIR, as shown in Figure 1a). At the same time, the space charge polarization effect can be enhanced by adsorbing more water molecules, leading to the rapid diffusion of 3DGF and the formation of protons between hydroxyl groups. [6]. To investigate the mechanism, band energy of graphene decorating with water molecule was theoretically simulated by density functional theory (DFT) in the Material Studio 8.0 software (Neotrident Technology Ltd. Beijing, China). Simply speaking, graphene is simulated by plane wave program implemented in CASTEP. Considering the single and double supercells (2 × 1 × 1 allowing edge reconstruction) under GGA-PBE with 9 × 1 × 1 k-points Monkhorst-Pack point grid and 500 eV plane wave base truncation, the graphene is simulated by plane wave program with basis cutoff of 500 eV. The geometry was optimized until the total energy reached 2 × 10^−5^ eV/atom and the maximum force acting on each atom is less than 0.05 eV/Å. For the 3D graphene foam and 3D graphene foam adding water molecule calculations, the CASTEP plane wave code was used under GGA-PBE considering a Monkhorst−Pack grid with 9 × 9 × 1 k-points and a plane wave basis cutoff of 500 eV; optimizing the geometry until the total energy reaches 2 × 10^−5^ eV/atom and the maximum force per atom exhibits values less than 0.05 eV/Å [30,31].

## 3. Results and Discussion

**Figure 3:**
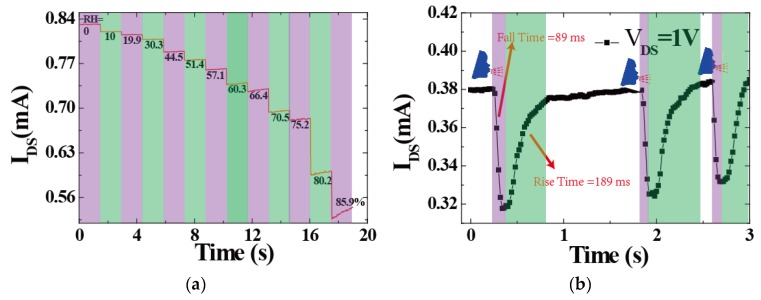
(**a**) Channel current response measurement of the 3DGF humidity sensor with varying different RH. (**b**) Response and recovery times of the device at 85% RH and the drain voltage was fixed at 1 V.

Furthermore, the humidity-sensing performance of the 3DGF sensors exposed to different RH levels (0%, 10.0%, 19.9%, 30.3%, 44.5%, 51.4%, 57.1%, 60.3%, 66.4%, 70.5%, 75.2%, 80.2%, and 85.9% RH) are presented in Figure 3a. In a closed air-tight box, the humidity sensors were measured by different RH values ranging from 0 to 85.9%. It can be seen that as the RH level increased, the obtained channel currents of the sensor reduced monotonically. To consider the real-time response and recovery times of the devices, the time-dependent response and recovery curves of the device to 85.9% RH are plotted in Figure 3b. The time taken by a sensor to achieve 85% RH of the total channel current was defined as the response or recovery time. The response and recovery times of the sensor were approximately 89 ms and 189 ms, respectively. Additionally, our humidity sensors exhibited reproducibility and long-term stability. Professionally, the hysteresis value is a vital parameter for humidity sensors as it determines the maximum time lag between the response time (adsorption process) and recovery time (desorption process). With respect to the water content in the environment, the hysteresis effect is defined by the difference between the resistances. In particular, for a perfect humidity sensor, the hysteresis value should be as small as possible or can even be negligible.

Table 1 compares the different characteristics of graphene-type humidity sensors including the response/recovery time, fabrication method, and sensitivity range. It was observed that the3DGF sensor exhibited broad sensitivity and rapid response and recovery rates.

**Figure 4:**
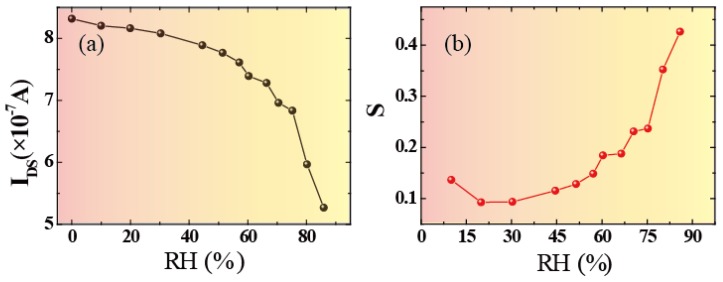
Relative humidity effect on the device performance. (**a**) Channel currents (*I_DS_*) with the relationship of RH (**b**) The variation in sensitivity of the device for different RH values.

It can be seen that our devices showed good uniformity. Quantitatively, the effect of relative humidity on the device is depicted in Figure 4. Figure 4a describes the relationship between channel current and relative humidity. It can be seen that the relationship showed a decreasing trend with the increase in water humidity. This also showed that the channel current (*I_DS_*) decreased more rapidly as relative humidity increased. To characterize the performance of the humidity sensor, the sensitivity (*S*) of the device was defined by Equation (1) [4,5,30,40]:

(1) $$S=\frac{\left|I_{wet}-I_{dry}\right|}{I_{dry}\mathrm{RH}}\times 100$$

where *I_wet_* and *I_dry_* represent the channel current of the device under wet and dry conditions (RH = 0%), respectively. As shown in Figure 4b, the sensitivity increased rapidly as RH increased. Due to its perfect performance, including its ultrafast response/recovery rate, our humidity sensors can be used for breathing monitoring or for developing new user interfaces (UIs). Figure 3b presents the ability of a 3DGF sensor to monitor human breathing. In particular, during the user’s speech, the ultrafast humidity sensor allowed the capture of fine features due to moisture modulation. Therefore, the 3DGF ultra-fast RH sensor can be used to identify different whistles, which can make use of low-cost and low-power sensors for user authentication.

**Figure 5:**
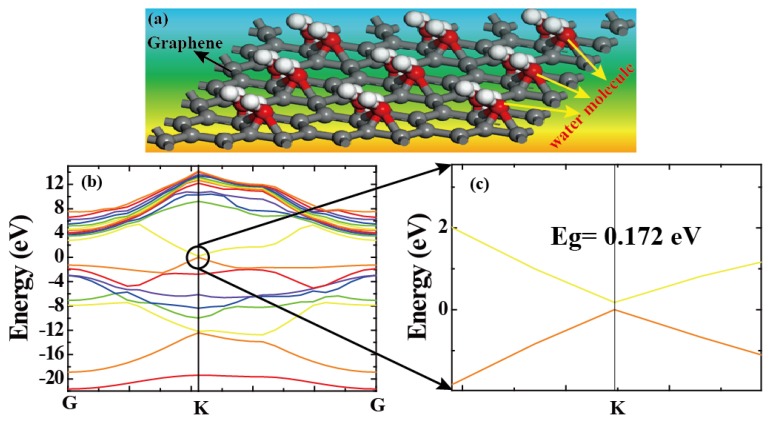
(**a**) The bonding mechanism between the graphene and water molecules. (**b**) The electronic band structure of graphene decorated with water. (**c**) The energy gap at the K point location.

A schematic model of humidity sensing at a 3DGF film is shown in Figure 5a. To investigate its mechanism, the band energy of graphene decorated with water molecules was theoretically simulated by density functional theory (DFT) in the Material Studio 8.0 software. As shown in Figure 5b, conductivity and valence are at K Brillouin point, which makes the material a direct bandgap semiconductor. The direct band gap at the K point was ~0.172 eV, as shown in Figure 5c. This can be ascribed to the donor effect [3] attributed to the donation of electrons from the chemically adsorbed water molecules to the 3DGF surface. The water molecules decorated in 3DGF will attract electrons. Simply, water molecules open the band gap of 3DGF. Meanwhile, electron density will decrease and the conduction level will rise, leading to the formation of band energy.

## 4. Conclusions

In summary, a three-dimensional graphene foam (3DGF) exhibiting super permeability to water was exploited in humidity sensors, enabling a humidity sensor with a broad range of % RH values and unprecedented response speed (response time: ~89 ms, recovery time: ~189 ms). The ultra-fast response speed of these sensors enables us to observe the regulation of moisture in a user’s breath. We constructed the 3DGF model decorated with water molecules theoretically and conducted the CASTEP as implemented in Materials Studio to calculate the energy structure. This allows sensors to be used in a variety of applications, such as humidity sensing, which we have experimentally verified with a cheap and easily available identification system. In addition, for different 3D materials, such as 3D transition metal dihalogenated hydrocarbons, ultra-thin nanoporous membranes for sensing applications can be realized in the interaction with different vapors and gases, which can be explored.

## Figures and Tables

**Table 1:** Comparison of different reported humidity sensors with graphene series materials.

Reference	Material	Sensing Range	Response/Recovery Time
Smith [30]	Graphene	1–96%	0.6 s/0.4 s
Ghosh [32]	Graphene	4–84%	180 s/180 s
Cai [33]	reduced graphene oxide (rGO)/graphene oxide (GO)/rGO	6.3–100%	1.9 s/3.9 s
Zhang [34]	Graphene oxide foam	36–92%	2 s/10 s
Trung [35]	rGO-polyurethane composites	10–70%	3.5 s/7 s
Leng [36]	GO/Nafion composite	11.3–97.3%	100–300 s/not shown
Bi [6]	GO	15–95%	10.5 s/41 s
Naik [37]	GO	30–95%	100 s/not shown
Yu [38]	GO/poly (sodium 4-styrenesulfonate) (PSS) composite	20–80%	60 s/50 s
Zhang [5]	rGO/poly(diallylimethyammonium chloride) PDDA composite	11–97%	108 s/94 s
Guo [39]	rGO	10–95%	50 s/3 s
This work	3DGF	0–85.9%	89 ms/189 ms
